# Spectroscopic Pulse Embeddings by Contrastive Learning from Unlabeled Data for Pile-Up Analysis

**DOI:** 10.3390/s26072138

**Published:** 2026-03-30

**Authors:** Congyu Lin, Xiaoying Zheng, Tom Trigano, Dima Bykhovsky, Yongxin Zhu, Li Tian

**Affiliations:** 1 Shanghai Advanced Research Institute, Chinese Academy of Sciences, Shanghai 201210, China; lincy@sari.ac.cn (C.L.);; 2 University of Chinese Academy of Sciences, Beijing 101408, China; 3 Electrical and Electronics Engineering Department, Sami Shamoon College of Engineering, Ashdod 7765181, Israel; 4 Electrical and Electronics Engineering Department, Shamoon College of Engineering, Be’er-Sheva 8410802, Israel

**Keywords:** contrastive learning, self-supervised learning, nuclear spectrometry, pile-up, representation learning

## Abstract

In nuclear spectroscopy, a physical phenomenon known as the pile-up effect distorts direct measurements by causing temporal overlap of detector pulses. Existing deep learning-based pile-up correction methods rely heavily on supervised training with simulated data, which often generalize poorly to real measurements due to simulation–experiment discrepancies. In this work, we propose a contrastive learning framework to learn robust and transferable representations directly from large-scale unlabeled real nuclear pulse signals. The detector output is segmented into physically complete pulse aggregations using a zero-crossing-based strategy, which serve as semantically coherent instances for representation learning. Physics-inspired data augmentations are designed to realistically model detector noise and bandwidth effects while preserving pulse area. A one-dimensional ResNet encoder is employed for efficient representation learning. The learned representations are transferred to pile-up identification and counting-rate estimation tasks. Experimental results on real nuclear radiation detection systems demonstrate that our method achieves strong performance and robustness under high counting-rate conditions, with particularly pronounced advantages in challenging peak pile-up scenarios.

## 1. Introduction

**Figure 1:**
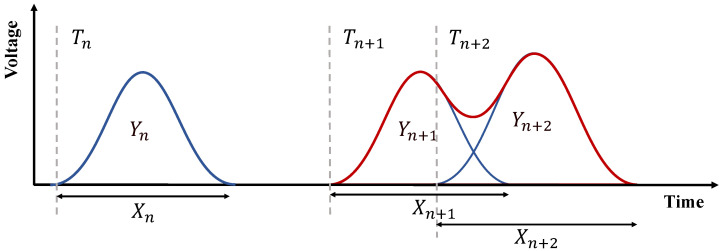
Illustration of pile-up: The input signal consists of photons with arrival times $T_{i}$, pulse durations $X_{i}$, and energies $Y_{i}$, where i=n,n+1,n+2. The blue curve represents the single pulses, and the red curve represents the pile-up pulse. Pile-up occurs when the (n+2)-th photon arrives during the busy period of the (n+1)-th photon.

Nuclear spectroscopy is widely applied in nuclear medicine, environmental monitoring, reactor diagnostics, and radioactive material characterization [1,2]. In a typical nuclear radiation detection system, the energy deposited by ionizing radiation in the detector material is converted into electrical signals. These signals are subsequently amplified, shaped, digitized, and analyzed to extract counting, energy, or timing information [3,4]. As illustrated in Figure 1, the pile-up phenomenon arises when successive photon arrivals occur within the duration of the electrical pulse produced by the front-end amplifier. The pile-up phenomenon distorts the pulse amplitude and deforms the waveform, thereby degrading the energy resolution, biasing activity estimation, and impairing subsequent signal analysis. This problem becomes increasingly pronounced at high counting rates [5,6].

The pile-up phenomenon has been extensively studied in nuclear spectroscopy. The most common approach is pile-up rejection, which detects overlapping pulses and discards them [7]. Although effective, this strategy inevitably prolongs experiments and can result in the loss of experimental data. Some methods attempt to compensate for rejected pulses through baseline subtraction [8], pulse clipping [9], or template fitting [10,11]. Other research has modeled the output signal as a linear combination of known pulse shapes, formulating pile-up as a sparse regression problem [12,13]. However, these approaches are generally most effective in systems with low pile-up probabilities and limited pulse-shape variability.

Moving beyond time-domain pulse recognition, recent studies have focused on analyzing collections of pulses. For instance, a nonlinear inversion approach was proposed for non-parametric pile-up correction [14]. Another work reformulated pile-up as a decompounding problem and employed a kernel-based estimator [15]. Similarly, the characteristic function of the energy spectrum has been exploited to infer the underlying photon energy distribution from indirect measurements [16]. However, these approaches typically require long data acquisition periods, which limits their applicability in real-time scenarios.

With recent breakthroughs in deep learning across various domains, nuclear spectroscopy has also benefited significantly from these advances. Convolutional neural networks (CNNs) have demonstrated strong capabilities in extracting pulse-shape features [17,18], while long short-term memory (LSTM) networks have been employed to capture temporal dependencies in pulse signals [19]. Transformer-based models have also been introduced to improve the resolution of nuclear spectroscopy [20]. More recently, Attn-UNet++, which integrates attention mechanisms into the U-Net architecture, has achieved remarkable performance on both general deep learning benchmarks and spectroscopy-specific evaluation metrics [21]. Furthermore, methods that jointly extract temporal and spatial features and explicitly incorporate count-rate information have been proposed to enhance robustness under high counting-rate conditions [22]. In contrast, SAEFit first fits an interpretable physical model to estimate target parameters, which are then used to reconstruct the signal, and has demonstrated strong performance in pile-up identification tasks [23].

**Figure 2:**
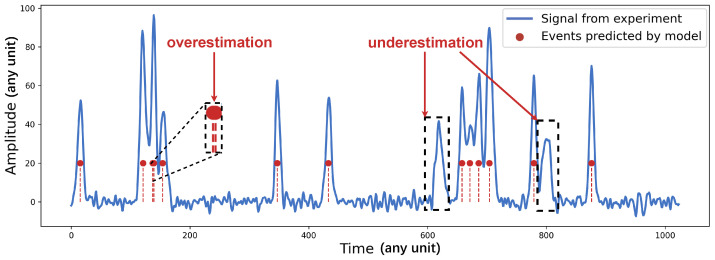
A fully supervised model trained on simulated data exhibits insufficient classification accuracy when applied to real experimental data.

However, under high counting-rate conditions, the accurate annotation of real nuclear pulse signals is often infeasible [19,21]. As a result, most existing deep learning-based pile-up correction methods are predominantly trained on simulated data. Although Monte Carlo simulations can reproduce key detector characteristics—such as charge-collection dynamics, shaped waveforms, and common noise sources—they are inherently imperfect. Uncertainties in signal models, approximations in physical processes, and unmodeled detector effects inevitably lead to discrepancies between simulated and experimental signals [24,25]. Figure 2 illustrates the performance of a model trained solely on simulated data when evaluated on real experimental measurements. As can be observed, severe deviations of experimental pulses from the idealized shapes assumed in simulation can result in significant estimation inaccuracies. These limitations highlight the urgent need for methods that can exploit large-scale unlabeled real experimental data to learn more generalizable feature representations.

Moreover, existing deep learning-based approaches are typically designed for supervised learning on a single downstream task, such as pile-up identification, counting-rate estimation, or energy spectrum reconstruction. However, these tasks are closely related and are likely to share a common underlying hidden representation of nuclear pulse signals. This observation motivates us to learn a general-purpose representation directly from large-scale real experimental data. The learned representation can be efficiently adapted to different downstream tasks through lightweight fine-tuning, thereby avoiding repetitive training of separate models for closely related tasks.

Contrastive learning is a self-supervised pretraining paradigm that learns meaningful representations by comparing different views of the same data sample without requiring manual annotations [26,27]. By bringing representations of positive sample pairs closer while separating those of negative pairs, the model can exploit large-scale unlabeled data to capture intrinsic structures and patterns, making it particularly suitable for scenarios where labeled data are scarce or difficult to obtain.

In this work, we introduce a contrastive learning framework to learn robust and transferable representations from large-scale unlabeled real nuclear pulse signals. Specifically, the detector output is first segmented into physically complete pulse aggregations using a zero-crossing-based method. These pulse aggregations serve as the fundamental units for representation learning. Based on these units, we design two physics-inspired data augmentation strategies that preserve essential physical invariants while increasing data diversity. This design enables semantically consistent contrastive learning. A one-dimensional ResNet [28] is employed as the encoder to balance representational capacity and computational efficiency. The learned representations are evaluated on pile-up identification and counting-rate estimation tasks, where they achieve consistently strong performance.

The main contributions of this work are summarized as follows:

- Contrastive learning framework for pile-up analysis: A contrastive learning framework is proposed for pile-up analysis, which leverages large-scale unlabeled real experimental data to learn robust and generalizable representations.
- Pulse-aggregation-level instance definition: A pulse-aggregation-level instance definition based on zero-crossing segmentation is introduced to ensure physically complete and semantically coherent training instances.
- Physics-inspired data augmentation: Physics-inspired data augmentation strategies are designed to model electronic noise and bandwidth effects while strictly preserving the pulse area, ensuring physically consistent invariances in the learned representations.
- Efficient encoder design: An efficient one-dimensional ResNet encoder is employed to achieve a balance between representational capacity and computational efficiency.
- Transferability and robustness: The learned representations generalize well across pile-up identification and counting-rate estimation tasks and maintain robustness under realistic deployment conditions.

## 2. Problem Formulation

### 2.1. Signal Model

The detector output can be theoretically modeled as a linear superposition of individual pulse responses [15]. In a digital acquisition system, the detector signal is represented in discrete time and can be expressed as

(1) $$X\left[n\right]=\sum_{k=1}^{\infty }\Phi_{k}\left[n-\lceil T_{k}\rceil \right]+\varepsilon \left[n\right],$$

where *n* denotes the discrete-time sampling index of the digitized signal, and $T_{k}$ represents the photon arrival times, which are assumed to form a sample path of a Poisson process [29]. Here, $\Phi_{k}$ denotes the *k*-th recorded electrical pulse, typically shaped into a quasi-Gaussian waveform by the front-end electronics. The term ε[n] represents additive electronic noise. The frequently used notations in this paper are summarized in Table 1.

### 2.2. Morphological Patterns

**Figure 3:**
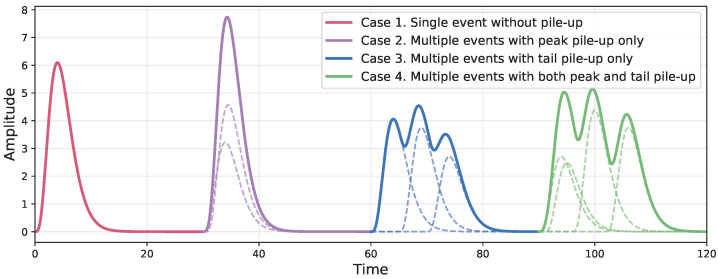
Visualization of four representative morphological patterns. The dashed lines represent the original pulses, and the solid lines represent the pulses affected by pile-up.

In pile-up theory, peak pile-up and tail pile-up refer to two distinct forms of waveform overlap [30]. Tail pile-up occurs when a subsequent pulse arrives on the falling tail of a preceding pulse, resulting in multiple distinct peaks within the same waveform. In contrast, peak pile-up occurs when pulses are so closely spaced in time that their peaks merge into a single unresolved maximum. Based on this distinction, as illustrated in Figure 3, the possible morphological patterns in detector signals can be categorized into four representative cases:

1. Single-event aggregations without pile-up;
2. Multiple-event aggregations exhibiting peak pile-up only (peak pile-up);
3. Multiple-event aggregations exhibiting tail pile-up only;
4. Multiple-event aggregations exhibiting both peak and tail pile-up.

Based on these definitions, two downstream tasks are considered and evaluated in this work, as follows.

#### 2.2.1. Pile-Up Identification Task

The first downstream task is pile-up identification, which determines whether a given pulse aggregation contains pile-up events. In practical applications, aggregations identified as pile-ups can be either discarded or further processed, depending on the analysis objective. Formally, this task is defined as a binary classification problem:

(2) $${\hat{y}}_{i}=g\left(S_{i}\right),$$

where ${\hat{y}}_{i}$ denotes the estimated pile-up label of pulse aggregation $S_{i}$, and g(·) represents the pile-up identification function. The true label $y_{i}$ for each pulse aggregation $S_{i}$ is defined as $y_{i}=1$ if the aggregation contains pile-up and $y_{i}=0$ otherwise.

Under ideal noise-free conditions, aggregations with multiple peaks (cases 3 and 4) can be easily identified as pile-up using peak-detection methods. However, single-peak aggregations corresponding to non-pile-up and peak pile-up (cases 1 and 2) are often difficult to distinguish. In realistic scenarios, electronic noise and waveform distortions further complicate pulse morphology, potentially causing misclassification even for multi-peak aggregations.

#### 2.2.2. Counting Rate Estimation Task

The second downstream task is counting rate estimation, which aims to estimate the number of photon events contained in a pulse aggregation. Formally, this task can be expressed as a multi-class estimation problem:

(3) $${\hat{y}}_{i}=f\left(S_{i}\right),$$

where ${\hat{y}}_{i}$ denotes the estimated number of events in pulse aggregation $S_{i}$, and f(·) represents the estimation function. The ground-truth event count $y_{i}$ is defined as the number of photon arrivals within the temporal interval of pulse aggregation $S_{i}$.

Under ideal conditions, single-event and tail pile-up aggregations (cases 1 and 3) can be accurately estimated using peak-detection approaches, whereas peak pile-up and combined peak–tail pile-up aggregations (cases 2 and 4) are prone to underestimation. In the presence of noise, peak distortion, and merging further complicate accurate counting rate estimation.

## 3. Methods

**Figure 4:**
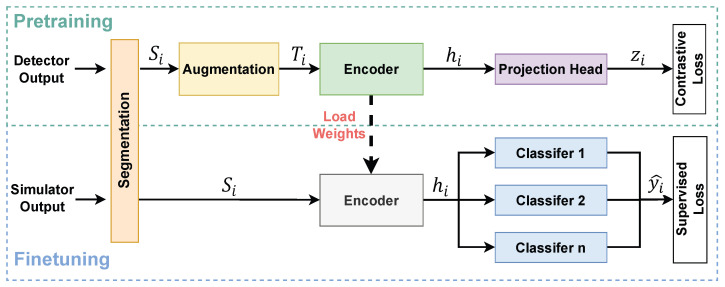
Workflow of the proposed method. In the pretraining stage, an encoder is trained on large-scale detector data and subsequently reused in the fine-tuning stage. During fine-tuning, the encoder extracts hidden representations, which are then fed into task-specific classifiers for different downstream tasks.

**Figure 5:**
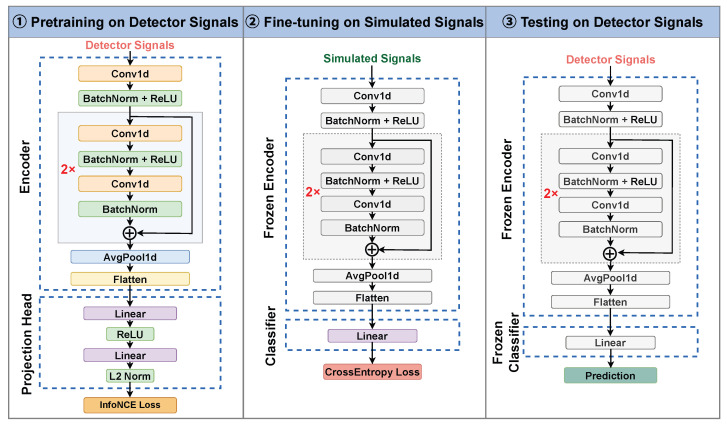
Detailed network architecture and parameter status across the three stages. The encoder and projection head are trained during pretraining. In the fine-tuning stage, the encoder is frozen and a task-specific classifier is trained. During testing, all parameters are fixed for inference. In this Figure, “2×” denotes that the same module is repeated twice.

As illustrated in Figure 4, during pretraining, the detector signals are first segmented and augmented. These augmented signals are then fed into an encoder equipped with a projection head. The encoder is trained using a contrastive objective, enabling the learning of robust signal representations. In the fine-tuning stage, the encoder loads the weights from the pretrained model. Task-specific classifiers are then trained on simulated data using a supervised loss. Figure 5 presents the detailed network architecture and the parameter update status in each stage. Table 2 summarizes the definitions and descriptions of common operations in the network.

### 3.1. Pulse-Aggregation-Level Instance Definition

Contrastive learning assumes that each training instance represents a semantically coherent entity. Its different augmented views should have the same underlying representation. Accordingly, an appropriate instance definition is required to ensure that each instance is associated with complete and unambiguous semantic content. In image classification [26,27], each image inherently corresponds to a well-defined instance. However, in nuclear pulse pile-up analysis, the continuous detector output lacks a natural instance boundary, necessitating a deliberate and principled instance formulation.

Conventional signal segmentation methods, such as fixed-length time-window segmentation and peak-centered fixed-length windowing, often ignore the physical boundaries of detector responses. As a result, these approaches may introduce artificial truncation or fragmentation of pulses, particularly under severe pile-up conditions. To address this issue, we adopt a zero-crossing-based segmentation strategy to partition the continuous detector output into multiple pulse aggregations. These pulse aggregations serve as the basic instances for contrastive representation learning

Specifically, given a digitized detector signal X[t], the baseline is first estimated from the pre-trigger samples and then subtracted from the signal, resulting in a zero-mean baseline. All discrete time indices $\left\{t_{i}\right\}$ satisfying the following condition are then identified:

(4) $$X\left[t_{i}\right]\cdot X\left[t_{i+1}\right]<0,$$

where $t_{i}$ and $t_{i+1}$ denote consecutive sampling instants. This condition indicates a change in signal polarity and corresponds to a zero-crossing event.

Based on the detected zero-crossing indices, the signal X[t] is partitioned into a set of disjoint pulse aggregations ${\left\{S_{i}\right\}}_{i=1}^{M}$, defined as

(5) $$S_{i}=\left\{X\left[t\right]|t\in \left[t_{i-1},t_{i}\right)\right\},\;i=1,2,\ldots ,M.$$

Here, $S_{i}$ denotes the *i*-th pulse aggregation, $t_{i-1}$ and $t_{i}$ are two consecutive zero-crossing indices, and *M* is the total number of aggregations obtained after segmentation.

To mitigate the influence of noise and ensure that only pulse aggregations associated with photon arrivals are retained, a subsequent thresholding operation *T* is applied. Formally, the threshold is defined as

(6) $$T=\mu_{\mathrm{noise}}+k\sigma_{\mathrm{noise}},$$

where $\mu_{\mathrm{noise}}$ and $\sigma_{\mathrm{noise}}$ denote the mean and standard deviation of the baseline noise, and *k* is a constant controlling the confidence level for noise rejection.

**Figure 6:**
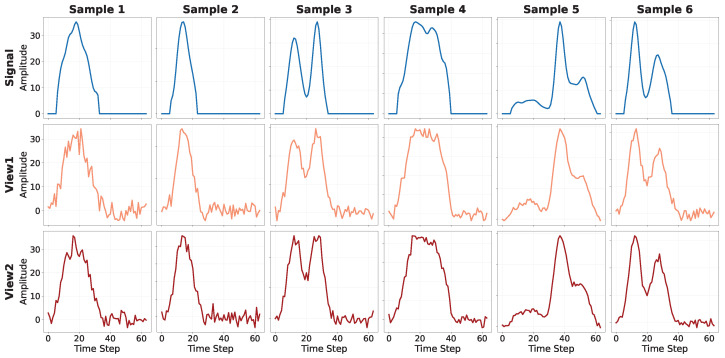
Visualization of physics-inspired data augmentation strategies. The first row shows the original pulse aggregations. The second and third rows present two augmented views generated through stochastic data augmentation. For each column, the samples in the second and third rows form a positive pair, while samples from different columns constitute negative pairs.

A pulse aggregation $S_{i}$ is retained only if $\max_{t\in [t_{i-1},t_{i})}|X\left[t\right]|>T.$ Pulse aggregations that do not satisfy this criterion are regarded as noise-only fluctuations and are therefore removed. Since the resulting segments have variable lengths, shorter segments are subsequently zero-padded to a fixed length of 64. This ensures that all segments have uniform dimensions, suitable for input to the neural network. The first row of Figure 6 presents representative examples of the segmented pulse aggregations.

The pulse aggregations obtained via zero-crossing are semantically coherent due to the following reasons:

1. Each pulse aggregation is strictly bounded by physical zero-crossings, which prevents artificial truncation of the signal and avoids introducing semantic ambiguity.
2. The amplitude-thresholding step further ensures that only genuine photon events are retained, excluding noise fluctuations.
3. Each aggregation contains a complete physical event—either a single pulse or a pile-up of overlapping pulses—preserving intrinsic physical information necessary for semantic coherence in contrastive learning.

### 3.2. Physics-Inspired Data Augmentation

In supervised learning, where the objective is to learn explicit class boundaries, data augmentation is used to increase the sample diversity. By contrast, contrastive learning aims to learn invariant features. In contrastive learning, data augmentation plays a central role in this process by constructing positive and negative sample pairs and implicitly defining semantic equivalence classes. As a result, the design of data augmentation directly determines the invariances captured by the learned representations.

For pile-up analysis, the proposed data augmentation strategies are designed to satisfy two key requirements. On the one hand, they are related to perturbations that may arise in practical radiation detection processes. On the other hand, they should preserve the pulse aggregation area as much as possible, which corresponds to the deposited photon energy [35].

Specifically, we first add zero-mean Gaussian noise to the original discrete signal to simulate electronic noise commonly present in nuclear pulse acquisition systems. Let X[n] denote the original signal; then, the noisy signal can be expressed as

(7) $$\tilde{X}\left[n\right]=X\left[n\right]+\eta \left[n\right],\;\eta \left[n\right]∼N\left(0,\sigma_{\eta }^{2}\right).$$

Subsequently, $\tilde{X}\left[n\right]$ is smoothed using a Gaussian convolution with an L1-normalized kernel G[k] of size 2K+1, which emulates the finite bandwidth of the detector electronics:

(8) $$X^{\prime }\left[n\right]=\sum_{k=-K}^{K}\tilde{X}\left[n-k\right]\;G\left[k\right],\;\sum_{k=-K}^{K}G\left[k\right]=1.$$

The total sum (or equivalently, the total pulse area) of the augmented signal can then be expressed as

(9) $$\sum_{n}X^{\prime }\left[n\right]=\sum_{n}\sum_{k=-K}^{K}\tilde{X}\left[n-k\right]\;G\left[k\right]=\sum_{k=-K}^{K}G\left[k\right]\sum_{n}\tilde{X}\left[n-k\right]=\sum_{n}\tilde{X}\left[n\right],$$

Since the additive noise has zero mean, i.e., E[η[n]]=0, it follows that

(10) $$E\left[\sum_{n}\tilde{X}\left[n\right]\right]=\sum_{n}X\left[n\right].$$

Therefore, the total sum (or area) of the augmented signal $X^{\prime }\left[n\right]$ is approximately equal to that of the original signal X[n], demonstrating that our augmentation strategy preserves the physical quantity of the total pulse area while introducing realistic fluctuations and shape variations for contrastive representation learning.

To illustrate the effects of these physics-inspired augmentations, Figure 6 visualizes example pulse aggregations. The first row shows the original pulse aggregations, while the second and third rows display two augmented views generated through stochastic data augmentation. The augmented pulse aggregations exhibit strong noise perturbations and varying degrees of smoothing, reflecting realistic variations in the signal. In each column, the samples in the second and third rows form a positive pair for contrastive learning, whereas samples from different columns are treated as negative pairs.

It is worth noting that the goal of the augmentation is not to replicate the precise spectral characteristics of the electronic noise but rather to introduce and deliberately amplify perturbations that challenge the learning process. This increases the difficulty of the contrastive task and encourages the encoder to learn more discriminative and robust signal representations. Consequently, the two data augmentation strategies described above can only simulate a subset of the possible noise sources.

### 3.3. Pretraining with Contrastive Learning

The primary objective of the pretraining stage is to learn a task-agnostic representation space that captures the intrinsic structure of the data. From an architectural perspective, the network consists of two main components: an encoder and a projection head. The encoder is responsible for extracting transferable and semantically meaningful representations from raw inputs, whereas the projection head maps these representations into a contrastive embedding space in which the contrastive objective is explicitly enforced.

**Figure 7:**
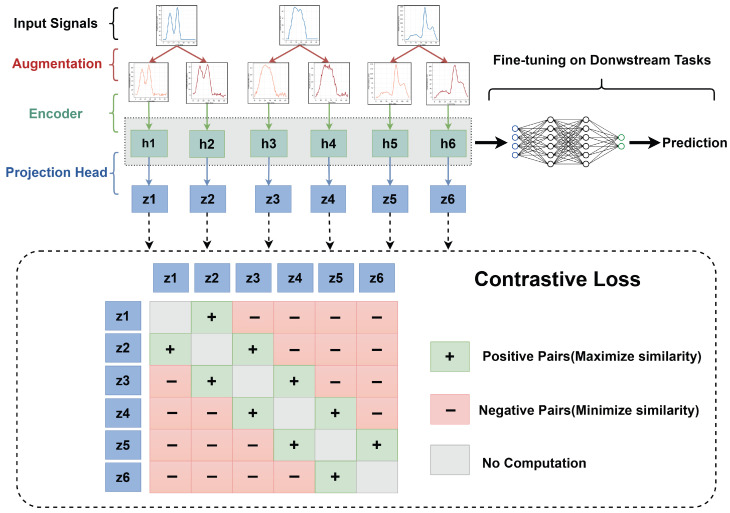
Data flow of the contrastive learning framework. The segmented signal $S_{i}$ is augmented to form $T_{i}$, which is encoded into latent representations $h_{i}$ by the encoder. These representations are projected to $z_{i}$ through a projection head and optimized using a contrastive loss. The pretrained encoder is then reused to produce hidden representations for task-specific downstream tasks.

Figure 7 depicts the overall data flow of the proposed contrastive learning method. Each segmented signal is first augmented to generate multiple views, which are then encoded into latent representations by the encoder. These representations are further mapped to a contrastive embedding space through a projection head and optimized using a contrastive loss. The pretrained encoder can subsequently be reused to extract hidden representations for task-specific downstream applications.

In our work, to balance representational capacity and computational efficiency, we adopt a shallow one-dimensional ResNet as the encoder. Specifically, as illustrated in Figure 5, the encoder consists of an initial convolutional layer followed by two residual basic blocks. Following the residual blocks, global average pooling and flattening are applied to obtain the final encoder output. Each residual basic block is composed of two convolutional layers with a skip connection, which can be formally expressed as

(11) $$f_{i}=f_{i-1}+\mathrm{Conv}\;\left(\mathrm{ReLU}\;\left(\mathrm{Conv}(f_{i-1})\right)\right),$$

where $f_{i-1}$ and $f_{i}$ denote the input and output feature maps of the *i*-th residual block, respectively. For clarity, batch normalization layers are omitted in the formulation but are applied after each convolution in practice.

The projection head is composed of two fully connected layers. Formally, it is defined as

(12) $$z_{i}=L2\mathrm{-norm}\;\left(\mathrm{Linear}\;\left(\mathrm{ReLU}\;\left(\mathrm{Linear}(h_{i})\right)\right)\right),$$

where L2-norm(·) normalizes the projected vector to unit length, thereby stabilizing training and ensuring that the cosine similarity employed in the contrastive loss is bounded within [−1,1].

It is noteworthy that the projection head is used exclusively during the pretraining phase, serving to decouple the encoder’s representation learning from the contrastive loss. This design ensures that the learned representations $h_{i}$ retain general discriminative features, while the projected vectors $z_{i}$ are optimized specifically for the contrastive objective.

The projected features are optimized using the InfoNCE loss:

(13) $$L_{CL}=-\log\frac{\exp\;\left(\mathrm{sim}(z_{i},z_{i}^{+})/\tau \right)}{\sum_{j}\exp\;\left(\mathrm{sim}(z_{i},z_{j})/\tau \right)},$$

where sim(·) denotes cosine similarity, and τ is a temperature parameter controlling the concentration of the similarity distribution. Here, $z_{i}$ and $z_{i}^{+}$ denote the projected representations of two augmented views derived from the same pulse signal, forming a positive pair. In contrast, $z_{j}$ represents projected features originating from different pulse signals and therefore constitutes negative pairs with $z_{i}$.

By minimizing the InfoNCE loss, the model is encouraged to maximize the similarity between positive pairs while simultaneously reducing the similarity between representations of different signals. Consequently, the encoder learns robust and discriminative representations that are invariant to stochastic data augmentations, providing a strong foundation for downstream nuclear spectrometry tasks.

### 3.4. Downstream Fine-Tuning

The primary objective of the fine-tuning stage is to adapt the learned generic representations to task-optimal discriminative capabilities. From an architectural perspective, the pretrained encoder is transferred to downstream tasks and used as a fixed feature extractor. All encoder parameters are frozen to preserve the representations learned from unlabeled real data. A task-specific classification head is appended and trained on labeled simulated data. This design facilitates effective domain transfer from simulation to real measurements.

For both pile-up identification and counting rate estimation, a lightweight single linear layer is adopted as the classification head. For pile-up identification, a binary cross-entropy (BCE) loss is employed:

(14) $$L_{\mathrm{BCE}}=-\frac{1}{N}\sum_{i=1}^{N}\left[y_{i}\log{\hat{y}}_{i}+\left(1-y_{i}\right)\log\left(1-{\hat{y}}_{i}\right)\right],$$

where $y_{i}\in \left\{0,1\right\}$ denotes the ground-truth label, and ${\hat{y}}_{i}$ is the predicted probability.

For counting rate estimation, the task is formulated as a multi-class classification problem, and the categorical cross-entropy (CE) loss is used:

(15) $$L_{\mathrm{CE}}=-\frac{1}{N}\sum_{i=1}^{N}\sum_{c=1}^{C}y_{i,c}\log{\hat{y}}_{i,c},$$

where *C* denotes the number of count-rate classes, $y_{i,c}\in \left\{0,1\right\}$ is the one-hot encoded label, and ${\hat{y}}_{i,c}$ represents the predicted probability for class *c*.

## 4. Dataset and Experiments Settings

### 4.1. Dataset Generation

In this paper, the dataset is divided into three subsets: a pretraining dataset, a fine-tuning dataset, and a test dataset. For the dataset derived from the Shanghai Synchrotron Radiation Facility (SSRF), both the pretraining and test datasets consist of detector signals acquired from X-ray fluorescence (XRF) experiments.

**Figure 8:**
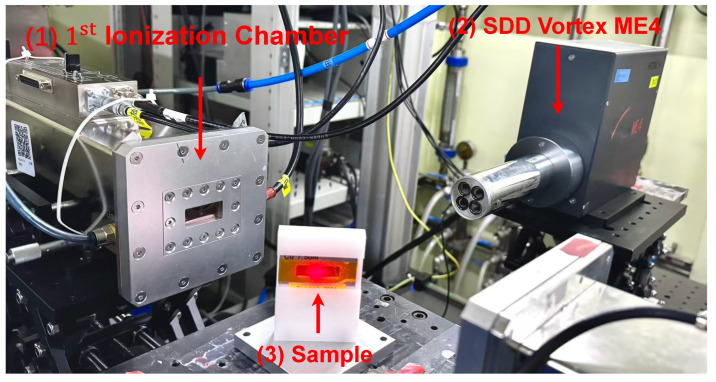
Diagram of the experimental setup at the SSRF beamline.

**Figure 9:**
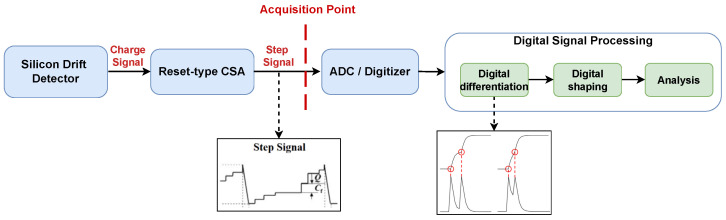
Diagram of data acquisition and processing in SSRF. The solid arrows represent the signal processing flow, while the dashed arrows indicate the signal state after specific processing steps. The red dashed line denotes the signal acquisition point.

A HITACHI Vortex-ME4 silicon drift detector (SDD), equipped with a reset-type charge-sensitive preamplifier, was employed for data acquisition. The signal-to-noise ratio is approximately 26dB. The digitization is performed immediately after the preamplifier stage, and all subsequent signal processing—including differential processing, pulse shaping, and pulse analysis—is implemented digitally. The same data acquisition and processing methodology is adopted as described in [20,21]. Figure 8 illustrates the data generation process of the XRF experiment, while Figure 9 shows the data acquisition and processing pipeline.

Measurements were conducted under three representative counting-rate conditions: 80 kcps, 450 kcps, and 1 Mcps. The pretraining dataset comprises samples of copper (Cu), cobalt (Co), and manganese (Mn) and records the entire energy spectrum for each element.

The fine-tuning dataset consists of simulated data generated using the Allpix Squared(version: 3.1.0) simulation framework [36]. Detailed simulation settings can be found in [37], and part of the simulation dataset is publicly available at https://github.com/Congyu-Lin/nuclear-pulse-dataset-for-pileup-correction (accessed on 3 January 2026). Eight representative elements, including Cu, Co, Ti, Mn, Mo, Se, Sc, and Zr, are simulated under different counting-rate conditions.

### 4.2. Annotation Strategy for Test Datasets

Real data cannot be labeled perfectly, which has long been a major challenge for applying machine learning methods to spectroscopy. In practice, no ideal real-data test set exists. To our knowledge, there are two main strategies for labeling real signals.

**Figure 10:**
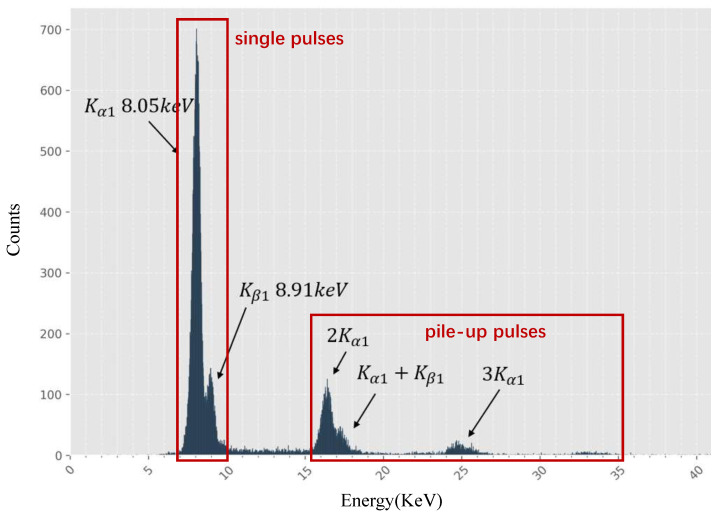
Cu spectrum at 1Mcps obtained via Pulse Area Analysis (PAA).

The first approach is to directly collect real pile-up signals. This method is feasible when the spectral energy range is relatively narrow, which implies that the energy distributions of single pulses and pile-up pulses do not significantly overlap, for example, in X-ray fluorescence (XRF) experiments involving elements such as Co, Fe, Cu, and Mn. In such cases, pulse areas can be analyzed and compared with the expected spectral distribution, while ambiguous pulses are discarded. Figure 10 shows the Cu XRF spectrum and labeling results at a counting rate of 1 Mcps obtained using Pulse Area Analysis (PAA). Because the characteristic energy range of Cu is approximately 7.5–9.5 keV, although the energy resolution of the PAA-derived spectrum is relatively low, we can still identify pulses with energies between 7–10 keV as single pulses and pulses above 15 keV as pile-up events. Pulses with energies between 10 keV and 15 keV are discarded as ambiguous. Statistics show that fewer than 0.3% of pulses are discarded at 1 Mcps and fewer than 0.1% at 450 kcps. Since this approach records real pile-up pulses directly, it can most faithfully reflect the true pile-up characteristics. However, reliable labeling is only possible in specific experimental scenarios, for certain elements, and within limited energy ranges. Therefore, the data produced in this way are mainly suitable for testing rather than for supervised training.

The second approach is to collect real single-pulse signals and then synthesize pile-up events by combining them. The advantage of this method is that it can generate a large amount of labeled data and is applicable to various elements and experimental conditions. However, such data remain synthetic and cannot fully reproduce the complex pile-up patterns and non-ideal noise present in real measurements. A representative example is the NSCL dataset, which is publicly available at https://github.com/sadeghi-bashir/SAEFit (accessed on 6 March 2026).

Therefore, in this work we use the dataset generated by the first approach (the SSRF dataset) to validate the fidelity of real pile-up signals and the dataset generated by the second approach (the NSCL dataset) to evaluate the generalization capability across different elements and experimental scenarios.

### 4.3. Training Parameters

For model training, the pretraining dataset contains 80,000 samples, while the fine-tuning and testing datasets consist of 10,000 and 2000 samples, respectively. A batch size of 64 is used for both pretraining and fine-tuning, and all models are trained for 30 epochs. The Adam optimizer is used with a learning rate of 0.001 and a weight decay of 1 × $10^{-4}$.

All experiments are implemented using the PyTorch (version: 2.3.1) framework, and training is conducted in parallel on two NVIDIA A30 GPUs. The full set of training parameters is summarized in Table 3.

### 4.4. Performance Metrics

In this work, pile-up identification and counting-rate estimation are regarded as independent tasks, with each evaluated according to its respective metric.

For pile-up identification, classification accuracy is adopted as the primary evaluation metric, defined as

(16) $$\mathrm{Accuracy}=\frac{\mathrm{TP}+\mathrm{TN}}{\mathrm{TP}+\mathrm{TN}+\mathrm{FP}+\mathrm{FN}},$$

where TP (true positive) and TN (true negative) denote correctly identified pile-up and non-pile-up events, respectively, while FP (false positive) and FN (false negative) correspond to misclassified samples. The mean accuracy and standard deviation over 100 fine-tuning runs are reported to assess model stability.

The performance of counting rate estimation is evaluated using the ratio between the output count rate (OCR) and the input count rate (ICR). Here, OCR denotes the total counting rate estimated by the model for a given pulse aggregation and is defined as

(17) $$\mathrm{OCR}=\sum_{i}{\hat{y}}_{i},$$

where ${\hat{y}}_{i}$ represents the estimated number of events in the *i*-th aggregation. In contrast, ICR corresponds to the known total counting rate received by the detector. An ideal counting system exhibits a linear response, characterized by OCR/ICR=1. Deviations from unity indicate underestimation (OCR/ICR<1) or overestimation (OCR/ICR>1) of the true counting rate.

## 5. Experiments and Evaluation

To comprehensively assess the effectiveness, robustness, and practical applicability of the proposed methods, a series of experiments are conducted from multiple perspectives. We first evaluate the core task performance, including pile-up identification accuracy and counting rate estimation, followed by a statistical significance analysis to ensure the reliability of the observed improvements. To further examine model robustness, we investigate generalization across diverse experimental scenarios and analyze the sim-to-real domain adaptation capability. In addition, we provide insights into the learned representations through visualization, and conduct extensive ablation studies to quantify the contribution of each component, including the impact of data augmentation strategies. Computational efficiency is also analyzed to assess the feasibility of real-time or large-scale deployment. Finally, we discuss the influence of varying experimental conditions and electronic system differences on model performance, highlighting robustness and potential limitations. Supplementary investigations on contrastive pretraining optimization (see Appendix A) and operational boundaries, including failure modes (see Appendix B), are provided in the appendices.

### 5.1. Pile-Up Identification Performance

In this subsection, we evaluate the pile-up identification performance of the proposed method. First, a comparative study with representative approaches is conducted under different counting-rate conditions. Then, misclassified pulse samples are analyzed in the time domain to investigate the main error patterns. Finally, a focused analysis on peak pile-up events is performed to reveal their temporal characteristics and inherent challenges.

#### 5.1.1. Comparative Evaluation with Representative Methods

In order to comprehensively compare the effectiveness of different learning paradigms and network architectures, three representative approaches are considered: (i) a fully supervised learning method based on a CNN encoder [17], (ii) a contrastive self-supervised learning method employing the same CNN encoder, and (iii) a contrastive self-supervised learning method with a ResNet encoder, corresponding to the proposed approach.

The performance of these methods is evaluated under three representative counting-rate conditions, namely 80 kcps, 450 kcps, and 1 Mcps, covering low, intermediate, and high counting-rate regimes. To assess the model generalizability, experiments are further conducted on real experimental data acquired using copper (Cu) and cobalt (Co) radiation sources. For each method, the mean classification accuracy and the corresponding standard deviation are computed from 100 independent fine-tuning runs on the downstream pile-up identification task.

**Figure 11:**
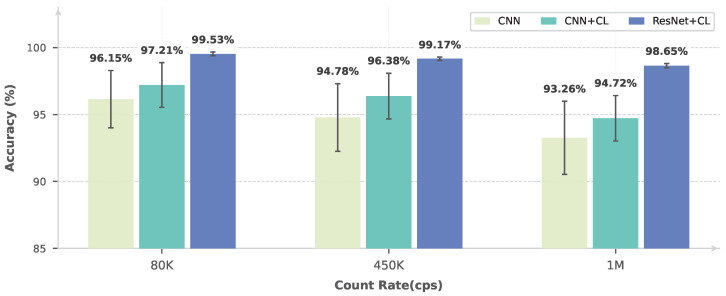
Pile-up identification performance for Cu under different counting-rate conditions.

**Figure 12:**
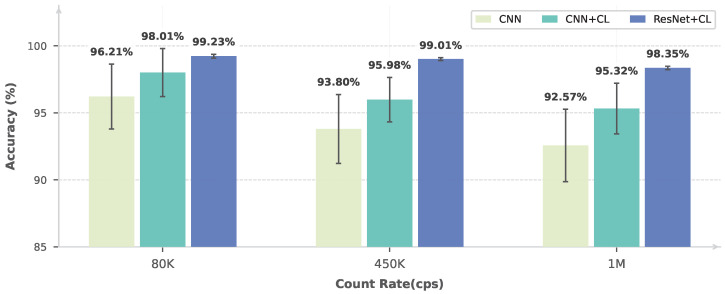
Pile-up identification performance for Co under different counting-rate conditions.

As shown in Figure 11 and Figure 12, the proposed ResNet-based self-supervised method consistently achieves the best performance across all counting-rate conditions, with a classification accuracy exceeding 98% and a standard deviation below 0.2%. The same performance trend is observed on both the Cu and Co datasets, indicating robust generalization across different radiation sources. These results demonstrate a clear advantage of the proposed method in terms of both accuracy and stability.

In addition, the contrastive learning approach based on the CNN encoder consistently outperforms the fully supervised CNN baseline, achieving higher mean accuracy and reduced performance variance. This comparison not only validates the effectiveness of self-supervised contrastive learning for pile-up identification but also highlights that the ResNet architecture provides stronger representational capacity for high counting-rate nuclear pulse analysis.

#### 5.1.2. Detailed Analysis of Misclassified Samples

**Figure 13:**
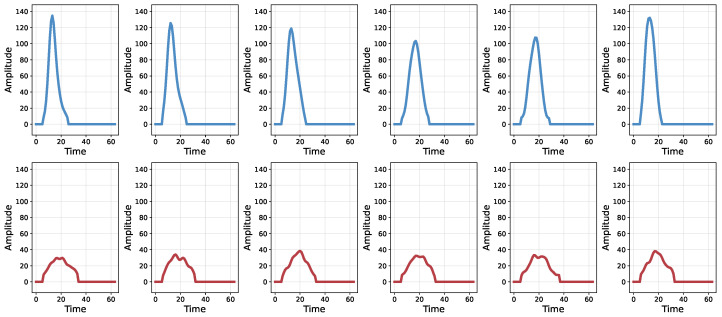
Typical misclassified real pulse examples in pile-up identification. The first row shows pile-up pulses misclassified as non-pile-up, while the second row shows non-pile-up pulses misclassified as pile-up.

To further investigate the characteristics and limitations of the proposed model, we analyze the misclassified pulse samples in detail. Figure 13 presents representative examples of incorrectly classified pulses. The first row corresponds to false negatives, where pile-up pulses are incorrectly identified as non-pile-up events, while the second row shows false positives, where non-pile-up pulses are misclassified as pile-up events.

We observe that most false negatives arise from peak pile-up scenarios, in which overlapping pulses exhibit waveform shapes that closely resemble those of single pulses, making them inherently difficult to distinguish. This observation is consistent with the theoretical analysis presented in Section 2.2.1, where Case 1 (single event) and Case 2 (peak pile-up only) are shown to be intrinsically ambiguous, representing a fundamental limitation of pile-up discrimination based on waveform morphology.

In contrast, false positives predominantly occur in the low-energy (low-amplitude) region, where the signal-to-noise ratio is relatively low. In such cases, noise superimposed on the original pulse introduces strong waveform perturbations that mimic pile-up characteristics, thereby increasing the difficulty of accurate classification. This result further highlights that, in practical nuclear radiation detection systems, the presence of various noise poses an additional challenge to reliable pile-up identification.

#### 5.1.3. Detailed Analysis of Peak Pile-Up Events

Peak pile-up (Section 2.2) has long been recognized as one of the most challenging cases in pile-up identification [30,38]. The experimental results presented in Section 5.1.2 further corroborate this observation. To specifically assess model performance under this challenging condition, we select peak pile-up samples of Cu elements acquired at a count rate of 1 Mcps and evaluate three different methods, as summarized in Table 4.

As can be observed, both the classification accuracy and stability degrade for all methods when confronted with peak pile-up events. Notably, however, the performance gap between the proposed method and the other two baselines becomes more pronounced under peak pile-up conditions. This indicates that the proposed method exhibits superior robustness and discriminative capability when handling highly overlapped pulses, particularly in challenging peak pile-up scenarios.

**Figure 14:**
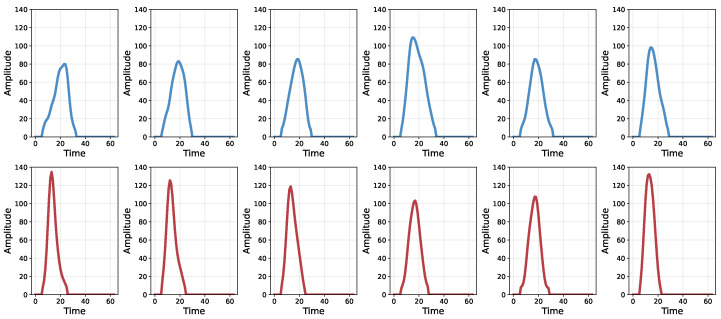
Temporal analysis of peak pile-up cases. The first row shows real peak pile-up pulses that are correctly classified by the proposed method, whereas the second row presents representative real peak pile-up pulses that are incorrectly classified.

To gain deeper insight, we further analyze which types of peak pile-up events can be successfully identified by the proposed model and which remain challenging. As shown in Figure 14, the first row presents representative peak pile-up events that are correctly identified by our method, while the second row shows misclassified examples.

It can be observed that the successfully identified peak pile-up pulses generally exhibit a larger full width at half maximum (FWHM), which is a standard and well-defined metric for characterizing pulse shapes. Specifically, the FWHM is defined as the temporal width of the pulse measured at 50% of the peak amplitude. Statistical analysis shows that the mean FWHM of correctly predicted samples is 12.01 sampling points, whereas the mean FWHM of misclassified samples is 8.84 sampling points.

From a physical perspective, a larger FWHM typically implies a shorter photon arrival interval Δt, defined as

(18) $$\Delta t=|t_{1}-t_{2}|,$$

where $t_{1}$ and $t_{2}$ denote the arrival times of two consecutive pulses. A smaller Δt corresponds to stronger overlap between pulses, whereas a larger Δt indicates that the pulses are more clearly separated.

**Figure 15:**
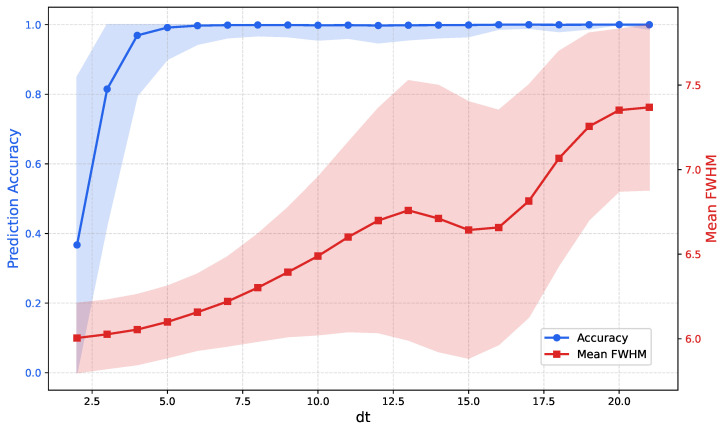
Relationship between pulse arrival time interval (Δt), FWHM, and classification accuracy. The solid lines represent the mean values, and the shaded areas represent the standard deviation.

To further investigate this relationship, we quantified the correlation between the pulse arrival interval (Δt), FWHM, and classification accuracy. Since the true arrival times of the sub-pulses forming pile-up signals are not available in the SSRF dataset, this analysis was conducted using synthesized pile-up data generated from single pulses in the NSCL dataset. As shown in Figure 15, a positive correlation is observed between the pulse arrival interval and both FWHM and classification accuracy, indicating that larger temporal separations facilitate more accurate pulse discrimination.

These results suggest that the proposed method can correctly identify the majority of peak pile-up events. However, pile-up cases with extremely short photon arrival intervals remain intrinsically difficult to resolve for methods relying solely on waveform morphology.

### 5.2. Counting Rate Estimation Performance

To verify that the representations learned through contrastive learning can be effectively transferred to different downstream tasks, we further evaluate the capability of counting rate estimation under various pile-up conditions. The output count rate (OCR) is examined under three representative input count rate (ICR) conditions, namely 80 kcps, 450 kcps, and 1 Mcps. The experiments are conducted on both copper (Cu) and cobalt (Co) radiation sources. The counting rate estimation performance of the three methods introduced in the previous section and Attn-UNet++ [21] is compared by plotting the OCR–ICR curves.

**Figure 16:**
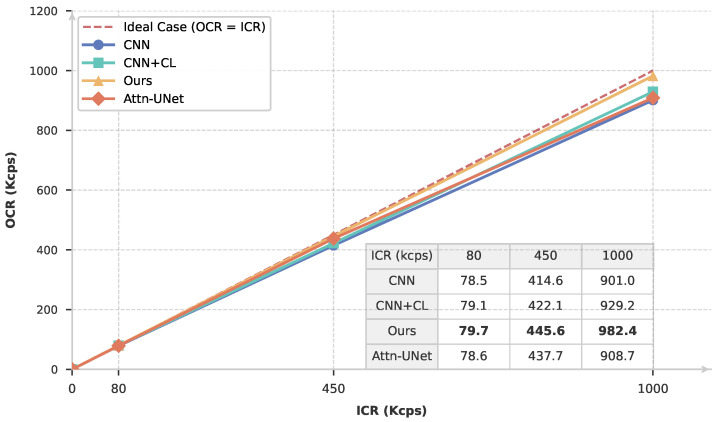
Counting rate estimation results for Cu under different counting-rate conditions. Different colors represent different estimation methods. The embedded table provides the detailed numerical results, where the bold values indicate the best performance at each counting rate.

**Figure 17:**
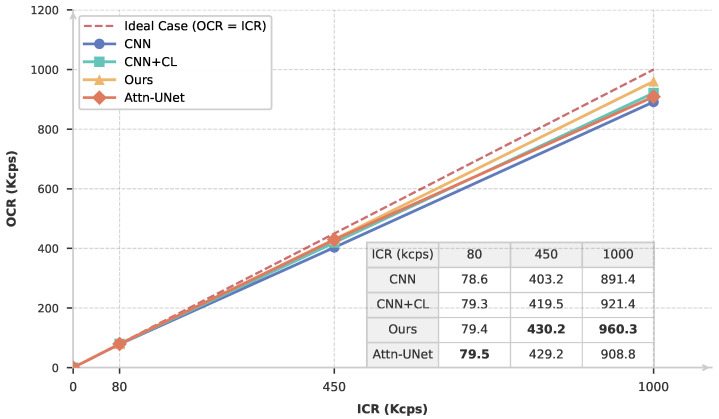
Counting rate estimation results for Co under different counting-rate conditions. Different colors represent different estimation methods. The embedded table provides the detailed numerical results, where the bold values indicate the best performance at each counting rate.

As shown in Figure 16 and Figure 17, at a low ICR of 80 kcps, where pile-up effects are relatively weak, all models exhibit satisfactory performance, with OCR values closely matching the corresponding ICR. However, as the counting rate increases to 450 kcps and further to 1 Mcps, clear performance differences begin to emerge. In particular, the proposed ResNet-based self-supervised method consistently produces OCR values that are closer to the true ICR than the other approaches. Although Attn-UNet++ is theoretically a powerful model for count-rate and energy spectrum estimation, the limitations of purely supervised learning methods become apparent in practical deployments with a significant sim-to-real gap. In particular, when the count rate increases to 1 Mcps, its performance can even fall below that of a simple CNN network trained with contrastive learning. The experimental results highlight the critical role of contrastive learning pretraining in improving accuracy and robustness.

In addition, a systematic performance difference is observed between the Cu and Co measurements. Overall, the counting rate estimation results obtained from Cu data are consistently superior to those from Co data. This behavior can be attributed to the difference in their characteristic X-ray energies: Co ($K_{\alpha }=6.93\;\mathrm{keV}$) emits lower-energy X-rays than Cu ($K_{\alpha }=8.05\;\mathrm{keV}$). Under identical detection conditions, the lower-energy Co signals suffer from a reduced signal-to-noise ratio, which degrades both pile-up discrimination and counting rate estimation accuracy. This effect becomes increasingly pronounced at high counting rates, where noise and pulse overlap jointly exacerbate estimation errors.

### 5.3. Statistical Significance Analysis

To demonstrate that the improvements of our method are statistically significant, we report the 95% confidence intervals (CIs) of multiple methods on the pile-up identification and count-rate estimation tasks. Each experiment was independently repeated 100 times with different random seeds. Additionally, paired *t*-tests were conducted, with each pair of experiments using the same random seed, for a total of 20 pairs.

Table 5 and Table 6 present the 95% CIs for the pile-up identification and count-rate estimation tasks, respectively, while Table 7 summarizes the results of the paired *t*-tests. The non-overlapping CIs and extremely small *p*-values indicate that the improvements of our method are statistically significant, suggesting that when applying deep learning to nuclear spectroscopy, sophisticated network architectures alone are insufficient, and it is crucial to directly address the characteristics of real data.

Furthermore, Table 7 clearly demonstrates that our representation learning approach can be easily adapted to different downstream tasks (both pile-up identification and counting rate estimation), a flexibility that task-specific methods such as Attn-UNet and SAEFit do not possess.

### 5.4. Generalization Across Different Experimental Scenarios

To evaluate the generalization capability of the proposed method across different detector types, measured particle species, experimental mechanisms, and signal characteristics, we further tested our model on an external dataset collected from a different experimental facility. The dataset is publicly available at https://github.com/sadeghi-bashir/SAEFit (accessed on 6 March 2026).

The SSRF experiments are based on X-ray fluorescence measurements. A silicon drift detector (SSD) is used to detect characteristic fluorescence signals from ground-state elements such as Cu, Co, and Mn. The signal-to-noise ratio (SNR) of these measurements is approximately 26 dB. In contrast, the external dataset originates from a decay spectroscopy experiment performed at the National Superconducting Cyclotron Laboratory (NSCL). The experiment uses a CeBr_3_ scintillation detector. This detector measures deposited energy from several radiation processes, including ion implantation events, β-decay electrons, γ rays, and other charged particles. The dataset has a significantly higher signal-to-noise ratio of approximately 65 dB. More details about the experimental setup can be found in Ref. [23].

Following the protocol in Ref. [23], the pretraining dataset contains 288,000 samples. The fine-tuning dataset contains 960,000 samples, and the test dataset contains 224,000 samples. The batch size is set to 1000. We adopt full-parameter fine-tuning during the fine-tuning stage. The model is pretrained for 30 epochs and fine-tuned for 400 epochs.

Table 8 summarizes the experimental results. Despite the substantial differences between the two experimental setups, the proposed method still achieves strong performance. This result demonstrates the robustness and generalization capability of our method across different detector systems, experimental modalities, and noise conditions.

### 5.5. Sim-to-Real Domain Adaptation

To quantitatively evaluate the effectiveness of the proposed method in mitigating the sim-to-real gap, we conducted a domain adaptation experiment on pile-up identification task. The simulated dataset was generated using Allpix Squared, while the real dataset was collected at SSRF at a count rate of 1 Mcps. To reveal the significant sim-to-real gap in nuclear radiation detection tasks, We first evaluated a conventional supervised learning approach under three training–testing settings: sim → sim, sim → real, and real → real. Next, we evaluated the domain adaptation method DANN under the sim→real setting. This provides a direct comparison between the proposed method and a representative domain adaptation baseline.

The experimental results are summarized in Table 9. As shown in the table, the conventional supervised method suffers from a substantial performance drop when transferring from simulated data to real data. This phenomenon has also been reported in previous studies [19,39]. In comparison, the proposed method significantly improves the performance under the sim → real setting and achieves better results than DANN [40]. This indicates that our method is more effective in reducing the domain discrepancy between simulated and real measurements.

It is worth noting that the model trained directly on the real dataset achieves the best performance. However, as discussed in Section 4.2, not all real experimental data can be reliably labeled in practice. Therefore, the real → real result only represents an ideal upper bound of the achievable performance and is not always feasible in real-world applications.

### 5.6. Representation Learning Visualization

To illustrate how the encoder organizes pulse aggregations according to their physical characteristics, we provide a UMAP visualization [41] of the learned representation space. The embeddings are color-coded according to the pile-up category.

**Figure 18:**
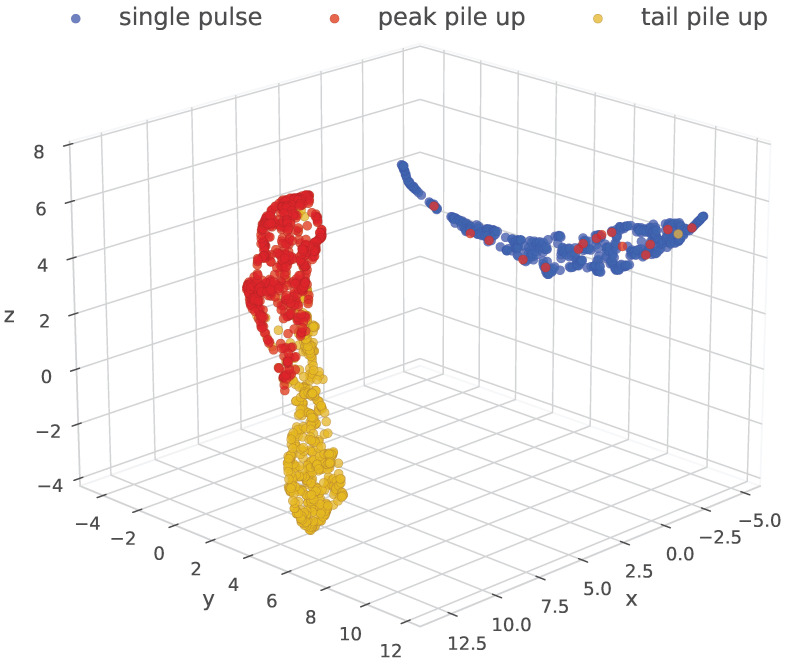
UMAP visualization of the learned pulse embeddings, color-coded by pile-up category. It shows that the encoder captures physically meaningful structures: single-pulse events are well separated from pile-up patterns, with peak pile-ups positioned closer to single pulses due to their morphological similarity.

As shown in Figure 18, the contrastive representations naturally separate single-pulse events from different types of pile-up patterns. Clear clusters can be observed for different categories. Interestingly, the degree of separation reflects the physical difficulty of each case. In particular, peak pile-up embeddings lie closer to the single-pulse cluster. This observation is consistent with the morphological similarity between peak pile-up events and single pulses, which makes them more difficult to distinguish.

### 5.7. Ablation Study

#### 5.7.1. Component-Wise Contribution Analysis

To quantitatively assess the contribution of each novel component, we conducted a series of ablation experiments. Specifically, we examined the effects of zero-crossing segmentation, Gaussian noise, Gaussian filtering, and contrastive learning pretraining on both pile-up identification and count rate estimation.

As reported in Table 10, contrastive learning pretraining plays a critical role when the distribution gap between simulated and real data is large, yielding the most significant improvement in overall performance. Zero-crossing segmentation, which enforces instance-level consistency during contrastive pretraining, provides the second largest contribution. Among the two data augmentation methods, Gaussian noise exhibits a stronger influence on performance than Gaussian filtering. Section 5.8 provides a more detailed analysis of how variations in these augmentations affect the algorithm’s behavior.

#### 5.7.2. Fine-Tuning Strategy Evaluation

We further evaluated the impact of different fine-tuning strategies on pile-up identification performance by comparing full-parameter fine-tuning with classifier-only fine-tuning. Interestingly, as shown in Table 11, classifier-only fine-tuning yields superior results on the SSRF test dataset, whereas full-parameter fine-tuning performs better on the NSCL test dataset. This phenomenon can be attributed to the difference in the sim–real domain gaps between the two datasets.

Specifically, the SSRF dataset was collected under relatively low signal-to-noise ratio (26 dB) conditions, while the NSCL dataset exhibits a higher SNR of 65 dB. The lower SNR in SSRF data introduces more challenging signal conditions. Moreover, as described in Section 4.2, SSRF data consist entirely of real signals, whereas NSCL data synthesize pile-up events using single-pulse real signals. Consequently, the SSRF test dataset contains more complex pile-up patterns and non-ideal noise present in real detector measurements. When the sim-to-real domain gap is large, classifier-only fine-tuning can better preserve pretrained representations and provide more stable generalization to the real data. Conversely, when the domain gap is small, full-parameter fine-tuning allows the network to fully adapt to the target domain, achieving higher performance. These observations suggest that the intrinsic signal quality and domain characteristics of each experimental scenario should guide the choice of the fine-tuning strategy.

### 5.8. Effect of Data Augmentation Parameters

**Figure 19:**
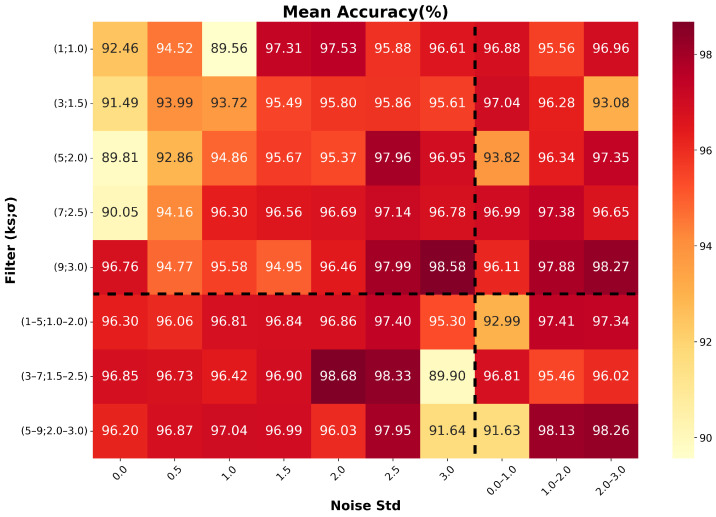
Mean pile-up identification accuracy as a function of data augmentation parameters.

**Figure 20:**
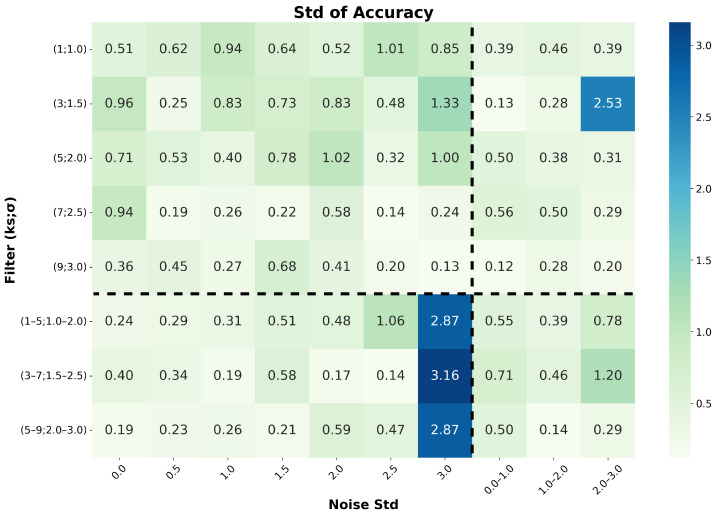
Standard deviation of pile-up identification accuracy as a function of data augmentation parameters.

To disentangle the contributions of individual augmentation strategies, we investigate the impact of the two data augmentation components on model accuracy and stability. The strength of additive Gaussian noise is controlled by varying the noise standard deviation, while the strength of Gaussian filtering is adjusted by changing the kernel size and the standard deviation of the Gaussian kernel. For both augmentation components, configurations with fixed parameters and randomly sampled parameters are evaluated, resulting in four distinct augmentation regimes. As illustrated in Figure 19 and Figure 20, the upper-left region corresponds to the case where both augmentation components use fixed parameters, while the lower-left region represents the configuration with fixed additive Gaussian noise and randomly sampled Gaussian filtering parameters.

From the upper-left regions of both figures, it can be observed that when fixed parameters are used, increasing the augmentation strength generally improves both the classification accuracy and stability. This indicates that, under fixed-parameter settings, stronger augmentations help the model learn more robust and discriminative representations by encouraging invariance to realistic signal perturbations.

Furthermore, the performance in the lower-left regions is predominantly superior to that in the upper-left regions across both figures. This indicates that, when the noise standard deviation is fixed, randomly varying the filtering parameters typically yields higher accuracy and improved stability.

This behavior can be attributed to differences in augmentation diversity. Although the additive Gaussian noise remains stochastic due to its inherent normal distribution even with a fixed standard deviation, fixed filtering parameters produce a limited set of smoothing patterns and thus restrict the sample diversity. In contrast, randomly sampling the filtering parameters introduces a wider range of signal variations, providing higher flexibility and leading to more effective representation learning. However, excessively large noise levels degrade performance and increase variability, suggesting that overly strong augmentations distort the intrinsic pulse morphology.

Overall, the best performance is achieved by combining a fixed intermediate noise standard deviation with randomly sampled filtering parameters within a moderate range. This configuration strikes an effective balance between augmentation strength and sample diversity, resulting in improved accuracy and enhanced training stability.

### 5.9. Computational Efficiency Analysis

To demonstrate the deployability of our method in practical applications, we conducted a computational complexity analysis and measured inference latency. We evaluated the latency of processing a single sample (consisting of 64 data points) in terms of FLOPs, number of parameters, and memory footprint, on both a server GPU (NVIDIA A30) and an embedded GPU (NVIDIA Jetson Orin Nano). The A30 environment was configured with Ubuntu 22.04 and PyTorch 2.8 + cu126 using Python 3.10, while the Jetson environment used JetPack 6.2.1 (CUDA 12.6) and PyTorch 2.5 with Python 3.10. The memory footprint was recorded using torch.cuda.max_memory_allocated(), and the results are summarized in Table 12.

Our method exhibits significantly lower time and space costs compared with other deep learning approaches in the nuclear spectroscopy domain, such as Attn-UNet++ and Transformer-based methods [20]. While the computational cost of our method is slightly higher than that of CNN-based models, previous analyses demonstrate that it achieves superior accuracy. The Transformer-based method [20,42] has already been deployed for real-time inference on FPGA. Considering that our method has even lower time and memory requirements, it is reasonable to expect that, with further optimizations such as quantization and pipelining, our approach could also achieve real-time inference.

### 5.10. Discussion of Experimental Conditions and Robustness to Electronics Variations

#### 5.10.1. Experimental Conditions and Representativeness

The signals used in this work were acquired from two representative experimental setups covering two major categories of radiation detection systems.

The first setup is representative of semiconductor detector systems with charge-sensitive preamplifiers and digital pulse processing. Specifically, the experiment was conducted at the X-ray fluorescence beamline of the Shanghai Synchrotron Radiation Facility (SSRF). A HITACHI Vortex-ME4 silicon drift detector (SDD) equipped with a reset-type charge-sensitive preamplifier was used. The charge signal generated by the detector was first amplified by the preamplifier and then directly digitized by an analog-to-digital converter (ADC) with a resolution of 16 bits and a sampling rate of 250 MHz. All subsequent signal processing, including differential processing, pulse shaping, and pulse analysis, was performed digitally. This configuration is representative of modern digital pulse processing systems used in X-ray spectroscopy.

The second experimental environment is representative of fast scintillation detector systems with a photomultiplier tube (PMT) readout. The experiment was conducted at the National Superconducting Cyclotron Laboratory (NSCL) in a decay spectroscopy setup [23]. A CeBr_3_ scintillation detector optically coupled to a 16 × 16 segmented Hamamatsu H13700 position-sensitive photomultiplier tube (PMT) was used. Radioactive ions were delivered to the detector by the A1900 fragment separator, where they were implanted and stopped inside the CeBr_3_ crystal. The detector system was connected to the NSCL digital data acquisition system (DDAS), which recorded waveform traces using Pixie-16 digitizers at a sampling rate of 250 MSPS. This setup is representative of fast timing scintillation detector systems widely used in nuclear physics experiments.

Together, these two setups cover two typical detector categories (semiconductor detectors and scintillation detectors) and two common front-end readout chains (charge-sensitive preamplifier readout and PMT-based readout), and therefore can be considered representative of a broad class of radiation detection systems.

#### 5.10.2. Robustness to Variations in Front-End Electronics

The robustness of the proposed method to variations in front-end electronics depends on the type of variation.

For baseline drift, the effect is inherently mitigated by the zero-crossing-based segmentation strategy used in this work. As described in Section 3.1, the baseline is estimated and subtracted from the signal before segmentation. This removes slow DC offsets and low-frequency baseline fluctuations, as long as the drift timescale is longer than a single pulse aggregation window. Therefore, the proposed method is inherently robust to baseline drift and does not require re-training or parameter re-tuning under typical baseline fluctuation conditions.

For reset effects, the reset-type preamplifier used in the SSRF setup introduces periodic reset transients in the raw step signal. As shown in Figure 9, these effects are handled in the differential processing stage, where the step signal is converted into shaped pulses. After differentiation, the waveform segments near the reset events exhibit waveform shapes that are significantly different from normal pulses and are therefore excluded during data preprocessing. Consequently, the learning model does not directly observe reset artifacts during training or inference, and the method is therefore robust to reset effects without requiring re-training or re-tuning.

For non-ideal noise, the robustness mainly originates from the representation learning strategy rather than from explicit noise modeling. The objective of contrastive learning is not to fit a specific noise distribution but to learn feature representations that remain invariant under signal perturbations. This invariance does not rely on whether the data augmentation exactly matches all possible non-ideal noise types. Instead, by training the model to produce consistent representations under a class of generic perturbations, the model is encouraged to focus on more stable physical attributes of the signal, such as pulse area and pulse aggregation structure, rather than high-frequency noise details. As a result, the model can generalize to more complex or non-Gaussian noise conditions encountered in real systems without requiring re-training or re-tuning.

However, if the front-end electronics differ significantly, such as a different pulse shaping architecture, substantially different shaping time, or a different detector type, the signal morphology may change noticeably. In such cases, re-training and re-tuning the model is necessary. This is consistent with most data-driven methods in nuclear instrumentation, where model performance depends on the similarity between training data and deployment conditions.

## 6. Conclusions

This work introduces a contrastive learning framework for robust representation learning in high counting-rate nuclear spectrometry. By leveraging large-scale unlabeled experimental data and physics-inspired data augmentation, the proposed method learns discriminative pulse representations that are resilient to severe pile-up effects. Extensive experiments demonstrate improved accuracy and stability in both pile-up identification and counting-rate estimation, with particularly strong performance in challenging peak pile-up scenarios, due to its ability to capture finer waveform morphological features.

It is worth noting that, although the proposed method demonstrates improved capability in peak pile-up analysis compared with existing approaches, peak pile-up cases with extremely short photon arrival intervals remain intrinsically difficult for waveform-morphology-based methods. Future work will therefore not be limited to temporal waveform analysis but may extend toward energy-spectrum-based representations to further address such extreme pile-up scenarios.

## Figures and Tables

**Table 1:** Summary of frequently used notations in this paper.

Notation	Definition
X[n]	Digitized detector output signal
$S_{i}$	Pulse aggregation segment
$T_{i}$	Augmented pulse aggregation
$h_{i}$	Hidden representation of pulse aggregation
$z_{i}$	Contrastive embedding vector
$z_{i}^{+}$	Positive embedding paired with $z_{i}$
$z_{j}$	Negative embedding paired with $z_{i}$
$y_{i}$	Ground-truth label
${\hat{y}}_{i}$	Model prediction

**Table 2:** Definitions of commonly used operations and functions in the network.

Operation	Description and Purpose	Formula/Example
ReLU (Rectified Linear Unit) [31]	Introduces nonlinearity to the network, enabling modeling of complex functions	f(x)=max(0,x)
sim(·) [26]	Cosine similarity between vectors; measures angular similarity and serves as a normalized correlation metric	$\mathrm{sim}\left(u,v\right)=\frac{u\cdot v}{{∥u∥}_{2}{∥v∥}_{2}}$
InfoNCE [32]	Contrastive loss that maximizes mutual information for augmented views of the same signal while minimizing it for different signals	$-\log\frac{\exp(\mathrm{sim}\left(z_{i},z_{j}\right)/\tau )}{\sum_{k}\exp\left(\mathrm{sim}\left(z_{i},z_{k}\right)/\tau \right)}$
AvgPool1D [33]	Global average pooling over the temporal dimension; compresses variable-length feature map into fixed-length representation	$y=\frac{1}{T}\sum_{t=1}^{T}x_{t}$
BatchNorm (Batch Normalization) [34]	Normalizes activations across mini-batch to zero mean and unit variance; stabilizes and accelerates training	$\hat{x}=\frac{x-\mu_{B}}{\sqrt{\sigma_{B}^{2}+\epsilon }}$
L2Norm (L2 Normalization) [26]	Scales vector to unit L2 norm; used to normalize feature embeddings before computing similarity	$v\leftarrow v/{∥v∥}_{2}$

**Table 3:** Training parameters on SSRF dataset.

Parameter	Value
Pretraining dataset size	80,000
Fine-tuning dataset size	10,000
testing dataset size	2000
Batch size for pretraining	64
Batch size for fine-tuning	64
Number of pretraining epochs	30
Number of fine-tuning epochs	30
Optimizer	Adam
Learning rate	0.001
Weight decay	1 × $10^{-4}$
Contrastive temperature τ	0.07

**Table 4:** Comparison of mean accuracy, standard deviation, and 95% confidence interval in peak pile-up identification across different methods, where results are averaged over 100 independent runs.

Method	Mean ± Std (%)	95% CI (%)
CNN	39.79 ± 15.93	[36.63, 42.95]
CNN + CL	64.15 ± 9.32	[62.30, 66.00]
ResNet + CL (Ours)	85.73 ± 2.26	[85.28, 86.18]

**Table 5:** Pile-up identification accuracy (%) and corresponding 95% confidence intervals under different elements and counting rates. The results are averaged over 100 independent runs.

Element	Input Count Rate	CNN	CNN + CL	ResNet + CL (Ours)
Cu	80 kcps	96.15 [95.73, 96.57]	97.21 [96.88, 97.54]	99.53 [99.50, 99.56]
Cu	450 kcps	94.78 [94.28, 95.28]	96.38 [96.04, 96.72]	99.17 [99.15, 99.19]
Cu	1 Mcps	93.26 [92.72, 93.80]	94.72 [94.38, 95.06]	98.65 [98.62, 98.68]
Co	80 kcps	96.21 [95.73, 96.69]	98.01 [97.65, 98.37]	99.23 [99.20, 99.26]
Co	450 kcps	93.80 [93.29, 94.31]	95.98 [95.65, 96.31]	99.01 [98.99, 99.03]
Co	1 Mcps	92.57 [92.03, 93.11]	95.32 [94.94, 95.70]	98.35 [98.32, 98.38]

**Table 6:** Mean counting rate estimation and corresponding 95% confidence intervals under different elements and count rates.

Element: ICR	CNN	CNN + CL	Attn-Unet++	ResNet + CL (Ours)
Cu: 80 kcps	78.46 [78.40, 78.52]	79.13 [79.09, 79.17]	79.64 [79.61, 79.67]	79.68 [79.66, 79.70]
Cu: 450 kcps	414.62 [413.62, 415.62]	422.10 [421.32, 422.88]	437.72 [436.91, 438.53]	445.65 [445.40, 445.90]
Cu: 1 Mcps	901.02 [899.83, 902.21]	929.19 [928.35, 930.03]	908.71 [907.72, 909.70]	982.36 [982.10, 982.62]
Co: 80 kcps	78.61 [78.55, 78.67]	79.31 [79.28, 79.34]	79.52 [79.49, 79.55]	79.38 [79.36, 79.40]
Co: 450 kcps	403.20 [401.99, 404.41]	419.47 [418.68, 420.26]	429.21 [428.24, 430.18]	430.21 [429.90, 430.52]
Co: 1 Mcps	891.43 [890.26, 892.60]	921.39 [920.73, 922.05]	901.75 [900.76, 902.74]	960.31 [960.06, 960.56]

**Table 7:** *p*-values obtained from paired *t*-tests comparing baseline methods with the proposed method (Ours) across different tasks. Smaller *p*-values indicate stronger statistical significance.

Method	Pile-Up Identification	Peak Pile-Up Identification	Counting Rate Estimation
CNN	$7.39\times 10^{-44}$	$7.58\times 10^{-68}$	$1.51\times 10^{-116}$
CNN + CL	$4.78\times 10^{-34}$	$9.12\times 10^{-43}$	$5.88\times 10^{-112}$
Attn-Unet++	–	–	$1.61\times 10^{-119}$
SAEFit	$6.58\times 10^{-55}$	–	–

**Table 8:** Comparison of pile-up identification accuracy between our method and SAEFit on the NSCL dataset.

Method	Mean ± Std	95% CI
SAEFit	99.63% ± 0.037%	[99.62%, 99.64%]
ResNet+CL (Ours)	99.81% ± 0.043%	[99.80%, 99.82%]

**Table 9:** Domain adaptation performance between simulated and real data.

Setting	Method	Accuracy
sim → sim	Supervised ResNet	99.91%
real → real	Supervised ResNet	99.07%
sim → real	Supervised ResNet	93.89%
sim → real	DANN	94.72%
sim → real	Ours	98.65%

**Table 10:** Ablation study of different components in the proposed method.

Method	Pile-Up Identification Accuracy	OCR in Count Rate Estimation
Full Method	98.56%	982.36
Replace zero-crossing with fixed length window	94.34%	927.11
w/o Gaussian noise	96.85%	930.04
w/o Gaussian filtering	97.53%	967.08
w/o Contrastive pretraining	93.40%	905.56

**Table 11:** Performance comparison between full-parameter fine-tuning and classifier-only fine-tuning on different datasets. Results are reported as accuracy (%).

Method	SSRF (26.2 dB)	NSCL (65.1 dB)
Full-Parameter	95.47	99.81
Classifier-only	98.65	95.29

**Table 12:** Computational complexity and inference efficiency comparison of different models on two hardware platforms: NVIDIA A30 GPU and NVIDIA Jetson. Latency is reported as mean ± standard deviation.

Method	FLOPs (M)	Params (M)	Memory Footprint (MiB)	Latency A30 (ms)	Latency Jetson (ms)
Ours	2.16	0.11	9.55	0.70 ± 0.01	2.82 ± 0.05
CNN	0.60	0.07	9.42	0.44 ± 0.01	1.70 ± 0.04
Attn-Unet++	13,314.29	119.1	494.83	21.91 ± 0.08	101.76 ± 1.71
Transformer	6510.61	6.35	494.14	5.66 ± 0.01	33.71 ± 0.90

**Figure A1:**
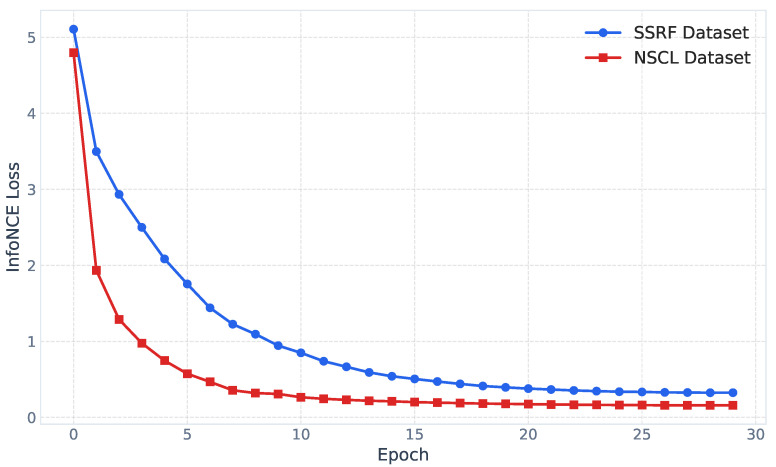
Pretraining loss curves of the contrastive learning process on the SSRF and NSCL datasets.

**Figure A2:**
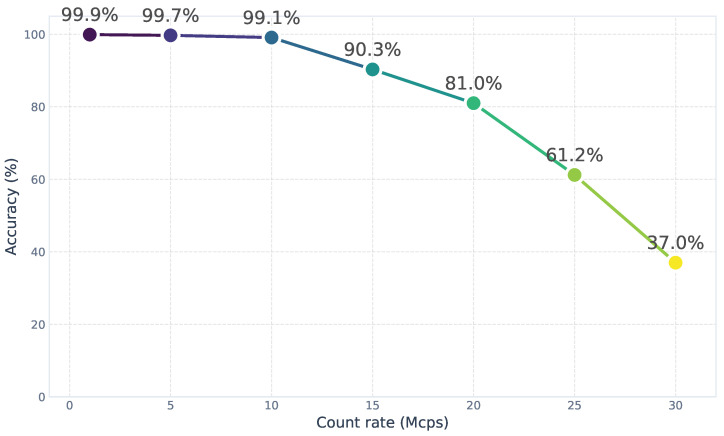
Pile-up identification accuracy as a function of counting rate on the NSCL dataset.

## Data Availability

The data presented in this study are available on request from the corresponding author. Access is restricted due to security constraints imposed by the data-providing laboratory and ongoing intellectual property development.

## Appendix A. Contrastive Pretraining Optimization

To analyze the convergence behavior of the contrastive learning process when applied to different nuclear pulse datasets, we present the training loss curves on both the SSRF and NSCL datasets. As shown in Figure A1, the loss decreases steadily and converges after 30 iterations, indicating that the proposed contrastive learning framework can be effectively optimized on both datasets.

## Appendix B. Operational Boundaries and Failure Modes

To evaluate the performance of the proposed method under extreme counting rates exceeding 1 Mcps and to determine its operational boundaries and failure modes, we conducted experiments on the NSCL dataset. As discussed earlier, this dataset allows the synthesis of pulse sequences with different counting rates.

We evaluated the pile-up identification accuracy across a wide range of counting rates. As illustrated in Figure A2, owing to the high signal-to-noise ratio (SNR) of the NSCL dataset, the proposed method maintains good performance when the counting rate is below 10 Mcps. However, the performance degrades rapidly once the counting rate exceeds 15 Mcps. When the counting rate reaches 30 Mcps, the prediction accuracy drops to below 40%.

Because the NSCL dataset exhibits a relatively high SNR (approximately 64 dB), it provides a favorable upper bound for analyzing the breakdown behavior of the algorithm. This implies that when the SNR of real experimental data is lower than that of the NSCL dataset, the proposed method is expected to fail at lower counting rates.
